# Adaptation across the Lifespan: Towards a Processual Evolutionary Explanation of Human Development

**DOI:** 10.1007/s12124-023-09767-y

**Published:** 2023-04-25

**Authors:** Werner Greve

**Affiliations:** Institute for Psychology, University of Hildesheim, Universitaetsplatz 1, 31141 Hildesheim, Germany

**Keywords:** Adaptation, Lifespan development, Evolutionary developmental psychology, Accommodative developmental regulation, Flexibility of goal adjustment, Theoretical interpretation & theory formulation

## Abstract

This paper argues that the project of a lifespan perspective in developmental psychology has not yet been systematically pursued. Overall, the number of age-specific papers far outweighs the number of lifespan approaches, and even approaches that focus on the lifespan as a whole are often restricted to adulthood. Further, there is a lack of approaches that examine cross-lifespan relationships. However, the lifespan perspective has brought with it a "processual turn" that suggests an examination of developmental regulatory processes that are either operative across the lifespan or develop across the lifespan. Accommodative adjustment of goals and evaluations in response to obstacles, loss, and threat is discussed as an example of such a process. Not only is it prototypical of efficacy and change of developmental regulation across the lifespan, but at the same time it makes clear that stability (e.g., of the self)—as a possible outcome of accommodation—is not an alternative to, but a variant of development. Explaining how accommodative adaptation changes, in turn, requires a broader perspective. For this purpose, an evolutionary approach to developmental psychology is proposed that not only views human development as a product of phylogenesis, but also applies the central concepts of the theory of evolution (adaptation and history) directly to ontogeny. The challenges, conditions, and limitations of such a theoretical application of adaptation to human development are discussed.

The time of grand theories in psychology, and especially in developmental psychology, is over, one might fear. Currently, an overarching theory of human development is not under discussion, especially not a theory that would explain why we develop: why at all, and why as we do. The last major paradigmatic attempt—the plea for the lifespan approach in developmental psychology—dates back more than three decades (Baltes, 1987). Actually, the expansion of the period of human change under consideration beyond childhood and adolescence was not fundamentally new even then; rather, it tied in with perspectives that had already been addressed several times at mid-century, for example by Charlotte Bühler, Erik H. Erikson, and Robert Havighurst (see, e.g., Lerner, 2018). But Baltes’ claims that development is not characterized by a specific direction, nor that can it be characterized as progress because gains and losses are discernible at all times, and that no life stage has any privileged importance for development, clearly went beyond the previous approaches.

In fact, the lifespan approach is not a revision of older theories focused on childhood and adolescence forced by empirical data (it had not escaped Freud or Piaget that people do change beyond adolescence), but a conceptual proposal to use the term “development” differently (i.e., more encompassing) than the classical understanding suggested (Freud and Piaget would not have *called* the changes during adulthood and especially older age “development”). The life span perspective of developmental psychology is, in a word, not a theory. Definitions, however, unlike theories, cannot be proven wrong. Whether the proposed use of the term ‘development’ (including also changes in adulthood) is fruitful and useful remains to be seen.

## The Lifespan Perspective on Human Development – A Project still Pending

For although the lifespan perspective has been generally approvingly received, and has inspired programmatic handbook contributions (prototypically: Baltes et al., 2006) and editions (e.g., Brandtstädter & Lindenberger, 2007; Fingerman et al., 2011; Lerner, 2010; Staudinger & Lindenberger, 2003), it is perhaps not unfair to sum up that these dedicated pleas have not succeeded in ensuring that developmental psychology actually focuses on the entire life span. In fact, approving references to this perspective (in the introductory paragraphs of books or articles) are often not much more than general statements: the vast majority of works in developmental psychology either deal with phenomena that are limited to individual life stages (e.g., first language acquisition or learning to walk) or with a specific developmental segment of a phenomenon that in principle also occurs in other developmental stages (e.g., emotion regulation in childhood). Reviews (e.g., Lansford, 2018, on well-being) or textbooks (e.g., Boyd & Bee, 2019) arguing in favor of a lifespan perspective just add sections or chapters on adulthood and old age, extending the time span for development, but do not integrate them. Relatively few investigations attempt to look at specific phenomena across the lifespan (for a summary, see also Moersdorf et al., 2022), for example, decision-making (Lindow & Lang, 2021), or theory of mind (Bernstein et al., 2011), or to examine methodological difficulties of this perspective (e.g., with respect to personality inventories: Brandt et al., 2020): not rarely, studies and paper still focus largely on adulthood (e.g., Heckhausen & Schulz, 1995; Heckhausen et al., 2010, 2019, on control) and thus do not investigate the entire lifespan.

The fact that a serious lifespan perspective has rarely been pursued is probably not so much because fundamental objections to the lifespan perspective (e.g., Bischof, 2008) are widely shared. Several methodological problems of the lifespan perspective (Moersdorf et al., 2022) are also unlikely to be insurmountable obstacles; in particular, problems of (ensuring) measurement equivalence across ages are not a problem specific to this perspective, but are also serious within shorter time periods in which considerable qualitative development occurs (cognitive development in childhood offers many examples of this; Bjorklund & Causey, 2018; Carey, 2009).

However, some conceptual problems of the lifespan approach have also not been sufficiently clarified (Moersdorf et al., 2022), which also affect the point discussed here. Perhaps the most far-reaching is the point raised at the outset that the lifespan approach is not a theory: it does not explain development to extend the time span considered (even if naming systematic changes across the lifespan as”development” were consensual). Rather, even studies that are explicitly committed to this approach add changes that occur after adolescence, if systematic enough, to the sequence of phases in the earlier stages of life (changes in cognitive abilities in older and high adulthood are a prototypical example; Baltes, Lindenberger & Staudinger, 2006; Baltes et al., 1999). However, at first glance these approaches hardly go beyond the (generalized) description of trajectories across life spans. Postulating a sequence of qualitatively distinguishable stages (across the life course) cannot explain development (Siegler, 1996). However, the processes that control and regulate—and thus explain—development are rarely named (Piaget is a notable exception: e.g., Chapman, 1988). In fact, phase-oriented approaches are not really sufficient even from a descriptive perspective: it has become increasingly evident that not only overtly culture-dependent phenomena such as morality or identity, but also phenomena that seem to be evolutionarily basal such as attachment are highly context-dependent (Keller et al., 2017).

It is therefore not surprising that the detailed study of individual objects and phenomena is more attractive, for instance the careful investigation of the timing, scope, and course of the ability of “theory of mind” (e.g., Bernstein et al., 2011; Derksen et al., 2018; Rakoczy, 2017; Rakoczy & Schmidt, 2013; Wellman et al., 2001), the physiological and neurological basis of changes in mental functions in old age (e.g., Lövdén et al., 2010), or the exact (micro-)course of emotional regulation processes (e.g., Blume et al., 2022). Notably, this research is certainly theory-driven, and it has often yielded not only empirical and theoretical insights, but also methodological advances (e.g., Neubauer et al., 2022). But its goal is typically a differentiated clarification of the respective focused phenomenon, rarely its placement in relation to other phenomena, and even more rarely a placement in a general picture of human development (Tomasello, 2019, is a notable exception).

## The Processual Turn: Developmental Regulation as a Unifying Perspective on Development

The lifespan approach comprises (although not entails) the assumption that the course of human development across the lifespan is not determined by a fixed or innate (e.g., genetical) program or blueprint. Human development is characterized by a high degree of plasticity (Baltes, 1987; Lövdén et al., 2010), it does not simply run along fixed steps, but its course depends on developmental conditions. In particular, modern lifespan approaches share the view that individual self-regulation of development becomes increasingly relevant from adolescence onward (Baltes, 1997; Brandtstädter, 2006; Brandtstädter & Lerner, 1999; Lerner & Busch-Rossnagel, 1981). Beyond universal trajectories and socio-cultural influences (Valsiner, 2000), the individual pursuit of developmental goals seem to play an essential shaping role: adult development is actively and intentionally shaped by the developing individual (Brandtstädter, 2006; Lerner & Busch-Rossnagel, 1981; Featherman & Lerner, 1985). In terms of universal descriptions of life courses, this complicates at first glance the quest for a unified theory of human development in adulthood (and thus across the life span): Life courses are characterized by a high degree of individuality (as Charlotte Bühler, 1933, had emphasized almost a century ago).

However, individuality does certainly not preclude a general, universal theory of human behavior and development (Valsiner, 2017). Actually, the actional explanation of human development marks the beginning of a change of the theoretical perspective. If “action” and the concepts implicit in it such as “goal”, “intention”, or “control”, do regulate human development (at least in adulthood) (Brandtstädter, 2006; Brandtstädter & Lerner, 1999), then this *is* a unified and cross-contextual construct relevant for the explanation of (individual) developmental trajectories. Accordingly, several lifespan approaches examined the role of goal and control for developmental trajectories (Brandtstädter, 1992; Freund et al., 2019; Freund & Riediger, 2006 Heckhausen et al., 2010, 2019).

Yet, certainly individuals cannot be “producers” but at best “co-producers” of their development: not only external constraints but in particular internal preconditions for action shape development no less than goals, intentions or decisions and goal-directed (development-related) action. Internal conditions or components of actions, however, cannot certainly not be explained in an action-theoretical format (Brandtstädter, 2006). Furthermore, the increase of problems and losses that particularly characterize late adulthood and old age in particular directed the attention to adaptation and compensation processes. Therefore, the role of compensatory and palliative regulatory processes has been a second important focus for developmental theories at the end of the twentieth century, perhaps even the more important one (Baltes, 1997; Baltes & Baltes, 1990; Brandtstädter, 2006; Brandtstädter & Greve, 1994; Brandtstädter & Renner, 1990; Brandtstädter & Rothermund, 2002; Freund, 2008; Freund & Baltes, 2000; Freund et al., 1999; Heckhausen et al., 2010, 2019; Staudinger & Lindenberger, 2003). The investigation of possible responses to challenges, blockages and losses in late adulthood and old age links the consideration of lifespan developmental trajectories to the (micro) processes of coping with current challenges: critical life events and current stresses as well as developmental tasks. One will hardly be able to contradict Popper's (1994) famous formulation “all life is problem solving” in this general form. Slightly exaggerated, one could subsequently say that it is the function of development to enable us to solve new problems. Since development itself consists of a sequence of challenges (problems, crises, tasks), problem solving is obviously constitutive for human development. Since individual development cannot be regulated independently of these micro-processes (precisely because it consists of them), conceptual links and equivalences between micro-processes and cross-cutting developmental trajectories are necessary prerequisites for any integrative perspective (Aldwin, 2007).

The argument by Baltes et al. (2006) to distinguish several theoretical levels (from macro- to micro-processes) asks for an integrative perspective that clarifies the inter-level-relationships. One of the difficulties of such integration is that while some responses to challenges and problems can be reconstructed in an intentional format (e.g., as “strategic responses”; e.g., compensatory efforts or systematic recruitment of social support)—by no means all of them. In particular, changing and adjusting goals in content or weight (letting go or devaluing and revaluing blocked goals, compensatory upweighting of alternative goals, etc.; Brandtstädter & Renner, 1990; Brandtstädter & Rothermund, 2002; Heckhausen et al., 2010, 2019; Wrosch et al., 2003a, b) typically does not occur intentionally and rarely in a controlled manner (Brandtstädter, 2006). The theoretical approaches mentioned discuss this conceptual tension between a personal (actional) response and a subpersonal (processual) regulation (Rothermund et al., 2020; Wentura, 2005) quite differently (Boerner & Jopp, 2007; Haase et al., 2013). However, for the purpose of the following considerations, such differences between these approaches (Kappes & Greve, subm.) are less significant than several common points.

To begin with, all of these theories of developmental regulation in adulthood and later life share a *processual* perspective: The focus is always on developmental regulatory processes (how are challenges and crises resolved?), not on identifying specific developmental phases or trajectories (who can, shows, or loses what and when?). Actually, this processual perspective is perhaps the more enduring yield of the lifespan perspective: Not so much a temporal expansion of the life stages and themes considered as primarily this “processual turn” in developmental psychology might become the most consequential aspect of the lifespan approach. This processual turn may not have been the main intention of these theories at first, and integrative potential of the process perspective has been too little exploited, but it does change the view of human ontogenesis.

For the purpose of the present argument, two aspects are particularly important. First, the view of regulative processes on human development opens the perspective to explain not only (continuous or discontinuous) changes, but also stability with reference to one and the same process—depending on the respective realized conditions and the level considered. Second, this opens the view of possible integration of developmental processes in childhood and adolescence that not just explain the basic conditions for later stages but rather provide necessary and constitutive components of adult regulatory processes. This might be a more promising way to an integrative lifespan perspective on human development.

### Stability: A Variant, not the Opposite of Development 

A number of studies have shown that the individual preparedness to adjust goals (flexibility of goal adjustment; Brandtstädter & Renner, 1990) is a predictor for the stability of the self and well-being (Brandtstädter & Greve, 1994; Brandtstädter et al., 1993) and a buffer against aversive circumstances and experiences (e.g., Greve et al., 2001; Greve & Enzmann, 2003; Greve et al., 2017; Rühs et al., 2017; Thomsen et al., 2015), and, thus, can explain unexpected patterns of findings of “lacking” changes (“well-being paradox”; Staudinger, 2000). The development of the self over the life span and especially the processes maintaining identity and continuity of the adult self (e.g., Brandtstädter & Greve, 1994; Greve & Wentura, 2003, 2010; Marek et al., 2022) can illustrate this thesis in a particular way, making clear how the processual view on development can explain different states (dynamically changing as well as appearing stable). Certainly, stability can arise in several ways. For example, stability and predictability are useful for social cooperation, and might therefore be intentionally pursued. Accordingly, the phenomenon of “cumulative stability” (Roberts & Caspi, 2003; Roberts & DelVecchio, 2000; Roberts et al., 2008) may be an expression of how our pursuit of stability is increasingly effectively realized in adulthood (Greve, 2005). But this shifts rather than answers the question: How can the stability of the self (and hence of personal goals) be explained, how does it come about, how do we maintain it—when so much seems to change in us and around us? How do we succeed in behaving and experiencing relative stability in changing environments and as life-long changing beings? For this, the reference to defensive mechanisms (overview: Leary & Tangney, 2012) is obviously not enough, because defensive mechanisms have costs: they fail to recognize realities that might sometimes be important to take note of. We need to know ourselves, especially our limits and weaknesses, perhaps not as precisely as possible, but as precisely as necessary, if our plans and actions are not to continually fail. Even compromising processes (e.g., “self-immunization”; Greve & Wentura, 2003, 2010) are insufficient because the experience of stability is then bought at the price of partial ignorance and decreasing social connectivity. We should register relevant physical changes in order to be able to react to them strategically (selective, optimizing, compensating: Freund et al., 1999). So we also need self-stabilization processes that accept realities, even unpleasant ones. This is precisely where the accommodative mode (Brandtstädter & Greve, 1994) comes in: The crucial point here is that accommodating (relative) importance does not imply denying change, but merely changing its meaning or value.

Hence, one of the major achievements of the processual view on human development is the insight that stability (at a certain level) can be considered a special case of development, not as the opposite; the apparent tension between change and stability can be resolved if attention is paid to which level and boundary conditions are in view in each case (Valsiner, 2000). At the same time, this allows for the integration of phenomena such as resilience, which only at first glance signal an alternative to development (under stressful conditions), but are actually the name for a (stable) developmental course under certain constellations of conditions (Greve & Staudinger, 2006; Leipold & Greve, 2009).

This perspective on stability as a developmental phenomenon (outcome) is the condition for the possibility of seriously applying the lifespan perspective to human development, because it overcomes (one can say: circumvents) the objection that adulthood is characterized by a high stability, that even tends to increase with age (Roberts & Caspi, 2003; Roberts & DelVecchio, 2000; Roberts et al., 2008). If stability were the opposite of development, then there would be no development in adulthood, and, thus, a lifespan perspective would be meaningless until very old age. If, however, stability (on a particular level) is seen as produced by processes on other (i.e.: lower) levels, if stability occurs *through* change (“allostasis”; Sterling & Eyer, 1988; see also Valsiner, 2000), stability becomes an *explanandum* of developmental theories. At the same time, this view avoids conceptual problems of explanations (of behavior or changes) that refer to stable attributes (traits, competencies, dispositions, etc.; Greve & Kappes, 2017).

### Produced Producer: Regulative Processes as Result of and Condition for Development

So far, the processual view leaves open the question of how developmental regulative processes develop: How and when they emerge and change, and why (Greve & Kappes, 2023). A number of studies has shown that individuals vary considerably with respect to their preparedness and ability to accommodate their goals or values (Brandtstädter, 2006; Brandtstädter & Rothermund, 2002; Haase et al., 2013; Heckhausen et al., 2010, 2019; Kappes & Greve, subm.). The fact that there are individual differences in the availability of these regulatory and adaptive processes indicates that they are dependent on developmental conditions and processes. Although this question remains unresolved for the time being, it is first important to note that accommodative and other adaptive processes are visible across the lifespan, not only across adulthood (Brandtstädter & Greve, 1994; Brandtstädter et al., 1993; Heckhausen et al., 2010, Heckhausen et al., 2019), but also in childhood and adolescence (Lessing et al., 2019; Thomsen & Greve, 2013; overview: Greve & Kappes, 2023). In this respect, accommodative processes are a plausible candidate for the assumption that developmental regulatory processes indeed regulate development across the lifespan.

Moreover, the example of accommodative developmental regulation can well illustrate how such a process can be both a product and a producer of development. In this context, it is helpful to note the distinction between “ontogenetic” and “deferred” adaptations (Bjorklund, 1997, 2021; see next section). It is worthwhile to distinguish between immediate functional forms of coping and regulation, on the one hand, and the developmental processes to be undergone necessarily in childhood and adolescence to build (the components of) adaptive forms of coping and regulation in adulthood, on the other. The examination of immediate (synchronous) age-related and prospective (diachronous) developmental functionality of the presence and change of a concrete capability will typically only allow an adequate explanation of the development of a capability such as accommodative developmental regulation when considered together.

Admittedly, the question of which processes regulate the development of the developmental regulatory process under consideration is theoretically complex, because the recognizable danger of a *regressus *ad infinitum can only be avoided if it were possible to identify a process (of the second order) that in turn does not require a further regulatory (third order) process for its explanation. It is a central concern of this contribution to sketch such a second-order (at the same time also first-order) process: adaptation. This, however, requires a second approach.

## Adaptation – An Evolutionary Perspective on Explanation of Developmental Dynamics

The second starting point is the insight that, in order to answer the question of why we develop at all (not all organisms do: bacteria, for example, do not), and why we develop as we do, an evolutionary perspective is necessary. Like all other phenomena involving humans (and all other living organisms), the fact of development itself, even more so the structural course of development are products of evolution. Especially human ontogenesis, which on the one hand enters the phase of reproduction extraordinarily late and on the other hand has a very extraordinarily long post-reproductive phase, seems to be in particular need of explanation from an evolutionary point of view.

### Evolutionary Psychology – The Developmental Gap of Evolutionary Theory Reiterated

However, an evolutionary perspective on psychology has been a long time coming: It was not until more than a decade after Wilson's seminal theses on “sociobiology” (1975) that the first work on evolutionary psychology attracted serious attention (an important step was the edition by Barkow et al., 1992). Meanwhile, there are studies on numerous areas of psychology (e.g., prosocial behavior: Nowak, 2006; antisocial behavior: Buss, 2005; Daly & Wilson, 1988; language: Pinker, 1994; intelligence: Geary, 2005; family: Salmon & Shackelford, 2007; personality Buss & Greiling, 1999), and a number of textbooks and editions have appeared (Badcock, 2000; Barrett et al., 2002; Buss, 2016, 2019; Crawford & Krebs, 1998; Dunbar & Barrett, 2009; Gaulin & McBurney, 2004; Laland & Brown, 2002; Palmer & Palmer, 2002; Plotkin, 2004; Workman & Reader, 2004). And there is much to suggest that this evolutionary perspective may actually be of help to psychology as a science (Zagaria et al., 2020).

Yet, in some respects the reception of an evolutionary perspective in psychology repeats the history of evolutionary theory in biology (Mayr, 1982): a developmental perspective received late and only marginal attention. Despite a few earlier precursors (Bonner, 1958; Gould, 1977), only evolutionary developmental biology (nicknamed "Evo-Devo"; Carroll, 2005) by the end of the twentieth century has made strong arguments that for evolutionary dynamics to be effective, not only reliable ontogeny, but precisely dynamic and adaptive individual development is at any rate useful, perhaps necessary (e.g., Arthur, 2011; Hall, 1999; West-Eberhard, 2003). Likewise, the developmental perspective in psychology has rarely been considered from an evolutionary theoretical perspective (despite early pioneers: J.M. Baldwin, 1902). Notable exceptions include in particular the seminal work of Bjorklund (1997, 2003, 2007, 2016, 2021; Björklund & Green, 1992; Bjorklund & Pellegrini, 2002; Geary & Björklund, 2000), among other contributions (e.g., Burgess & MacDonald, 2005; Konner, 2010: for review: Bjorklund et al., 2016). The perspective of evolutionary developmental psychology has earned the merit not only of pointing out the fact in the first place that human development, like other human attributes and processes, must be the product of evolution, but also of making convincingly clear how this insight can be used empirically to better understand human development.

Nevertheless, an evolutionary perspective on human development has remained a specialized field. Although some developmental psychology textbooks have also now incorporated this perspective (Greve & Bjorklund, 2018; by no means all of them – especially when the life span perspective is followed), and evolutionary psychology handbooks also have a chapter on development (Buss, 2016, 2019), neither developmental psychology textbooks have systematically linked the evolutionary perspective to other aspects (of development or human behavior) nor evolutionary psychological textbooks refer systematically to a developmental point of view (beyond adding it as one important aspect among others).

### Evolutionary Developmental Psychology: Is a Lifespan Perspective Compatible?

In particular, the lifespan perspective of developmental psychology has very rarely been linked to an evolutionary perspective (Greve & Bjorklund, 2009; see also Bogin, 1997; Bogin & Smith, 1996). Rather, work on evolutionary developmental psychology has focused almost exclusively on the first decade of life (Bjorklund, 2021; Bjorklund & Ellis, 2014). Although evolutionary theory formulated the question of an explanation of postreproductive old age early on (e.g., Austad, 1997; Carey & Judge, 2001), and anthropology has discussed, for example, the role of grandparents in reproduction in a number of studies (Hawkes, 2004; Voland et al., 2005), the viewpoint of age has hardly been discussed from an evolutionary perspective in developmental psychology (Greve & Bjorklund, 2009). In fact, at least at first glance, the life span perspective does not seem to fit with an evolutionary perspective: already the phenomenon of aging itself, but even more so a systematic structure of aging, can hardly be explained, it seems, for a life stage in which reproduction has already taken place: “The benefits resulting from evolutionary selection evince a negative age correlation” (Baltes, 1997, p. 367).

There are, however, god reasons for the compatibility of an evolutionary perspective with a lifespan perspective (Greve & Bjorklund, 2009), in particular also for a compatibility with stabilizing processes in general and accommodative processes in particular (Greve & Thomsen, 2016; Greve et al., 2014). If the functionality of older and old members of a community is to use the accumulated and integrated experiences of a (long) life to improve the survival and reproductive prospects of the younger members of the group (Diamond, 2001; Mergler & Goldstein, 1983)), then this requires not only communicative skills and transgenerational interactions (Kessler & Staudinger, 2007; Voland et al., 2005), also not only the ability to memorize (Mergler & Goldstein, 1983) and remember especially older experiences (Rubin et al., 1986), but also the ability to integrate single or regular experiences into a cognitive network that allows to give advice also for new, at best structurally comparable cases and constellations. Wisdom (defined as a well-balanced coordination of emotions, motivation, and thought, with good judgement and the ability to offer advice in difficult and uncertain matters of life; Staudinger & Dörner, 2006) should prove an important resource of the elderly (e.g., Baltes & Staudinger, 2000; Staudinger, 1999). Wisdom, and its usage for younger generations, in turn, apparently presupposes stability of numerous mental functions—from cognitive capacities to integrative functions of self and personality (such as processes of self-esteem maintenance). It is for this function that the processes of stabilization mentioned above are relevant, presumably necessary, especially in late adulthood and old age (Greve & Bjorklund, 2009).

### Adaptation: From Phylogeny to Ontogeny

However, even the extension of the evolutionary perspective to the whole life span (in particular, as mentioned above, the older age) does not use the theoretical potential of the evolutionary theory sufficiently. The special point of evolutionary theory is its abstraction. The process of adaptation (channeled through history, which documents and reflects the contingent factual course of the preceding phylogenesis) is the conceptual core of the theory (Gould, 2002; Maynard Smith, 1958). Note that this theoretical core is not dependent on concrete (physiological or biochemical) realization: Remarkably, Darwin's conception of how heredity works was entirely wrong, and remarkably, the concrete mechanism of organic heredity is entirely indifferent to the soundness of his theory. Yet the systematic application of the theoretical core of evolutionary theory – adaptation and history (Greve & Kappes, 2017) – to human ontogeny is still pending (for exceptions, see Lickliter & Honeycutt, 2003; Siegler, 1996). This concerns in particular the central concept of adaptation (“selective retention of fitness-enhancing variants”; Campbell, 1960, 1969)—and thus the concepts of selection, retention, fitness, and variation entailed by it.

Why is adaptation more than a prototypical example for an application of evolutionary concepts on human development? The main idea of this plea is that adaptation is a process that is also self-explanatory—at least that does not in turn require another process to explain it, thus avoiding infinite regress. One important line of reasoning leading to this argument is the consideration that adaptability, which varies between species, is itself a product of evolutionary and thus adaptive processes (e.g., Lasker, 1969; Moran, 2008; Wagner, 2005; Wagner & Altenberg, 1996). The analogous application of this idea to human development leads to an explanation of individual differences in adaptability – and thus back to individual differences with respect to adaptive regulatory processes (e.g., accommodation).

Accordingly, if individual adaptivity (both ontogenetical and micro-processual) is a core of (human) development, if adaptivity has developed throughout our phylogeny (i.e. evolution), and if both levels (“dimensions”) of development (phylogeny and ontogeny) proceed according to the very same principles (selective retention of fitness-enhancing variants), then individual development could be viewed as adaptation to “tasks” or “problems” or “crises”, and the ontogenetical development of individual adaptivity should follow these (adaptive) principles as well. According to this idea, adaptation would be, at the same time, process and product of development (on phylogenetical, ontogenetical and microprocessual layers).

One implication of this perspective is the assumption that development, like evolution, is stimulized or even forced by problems. If adaptivity is a developmental result of a variety of reactions to challenges, then individual adaptivity should benefit from a variety (heterogeneity) of challenges (problems, tasks, crises). There is some first evidence that heterogeneous life circumstances actually support (at least predict) individual adaptivity (here: individual preparedness and capability of goal accommodation). For instance, Greve and Thomsen (2013; Thomsen & Greve, 2013) found in two studies that heterogeneous leisure activities in childhood predict flexibility of goal adjustment in adolescence, and Greve et al. (2021) found that multilingualism in childhood predicts flexibility of goal adjustment in adulthood.

### Adaptation: Theoretical Challenges

The recursive ("self-explanatory") potential of the concept of adaptation addressed above is demonstrated by the fact that adaptation could (help) explain how differences in adaptivity arise. In a study of a group of genetically identical mice (Freund et al., 2013) raised in a large and heterogeneous cage environment, it was shown that initially random behavioral variations between individual mice self-sustained into differential behavioral dispositions that led to increasingly distinct differences in how they used the (potentially) heterogeneous cage environment. The differences in explorativeness (and thus heterogeneity of developmental stimulation) in turn then led to measurable developmental differences (in this study, brain structures). The unsystematic variation, which is at the beginning of the explanation, then leads to different manifest developmental trajectories via the different effects that the varying behavior has on the behaving individual. It is precisely this process that the concept of adaptation describes.

Thus, the central concern of the theoretical application of evolution in general and adaptation in particular is to investigate to what extent the dynamics (selection), consistency (retention), and individuality (variation) of human development can be described in a theoretically integrative way with the help of these concepts (among some others). To this end, several theoretical problems need to be discussed, including (i) the more precise conceptualization of adaptation (selection, variation, retention, fitness), (ii) the question of what other evolutionary concepts can or must be considered for a theoretical perspective on human development, and (iii) the question of the unit of (evolutionary) selection.

(i) Evolutionary theory has admittedly struggled with the centrality of the concept of adaptation since the seminal critique of Gould and Lewontin (1978). Actually, there are several competing conceptualizations (e.g., Andrews et al., 2003; Buller, 2005; Buss et al., 1998; Orzack & Sober, 2001; Reeve & Sherman, 1993; Rose & Lauder, 1996; Williams, 1966), in particular with respect to the question of whether everything we can observe at a given point in time in terms of distinctive (at least widespread) attributes is an adaptation (functional with respect to the given circumstances: Fisher, 1930; Reeve & Sherman, 1993), or the result of an adaptation (functional with respect to former circumstances: Sterelny & Griffiths, 1999), or both (Grafen, 1982; Williams, 1966).
  Of course, numerous approaches have considered human behavior as adaptation (e.g., Cronk et al., 2000). What needs to be clarified, therefore, is a viable notion of adaptation that can also be connectable in psychology: there are countless examples of a merely metaphorical use of the notion of adaptation, especially in psychology. Adaptation is now ubiquitous in numerous areas of psychology, not just in various strands of evolutionary psychology (Barkow et al., 1992; Bjorklund & Ellis, 2005; Buss et al., 1998), but in numerous other fields, such as emotion regulation (Lazarus, 1991), coping theories (Taylor, 1983; Vaillant, 1977; Wrosch et al., 2003a, b), personality approaches (Buss & Greiling, 1999), rationality theories (Gigerenzer, 2000), cognitive processes (Anderson, 1991), and, of course, human development (e.g., Baltes, 1997; Greve et al., 2005; Piaget, 1978, 1980). If we are to overcome a mere metaphorical (loose) usage of the concept of adaptation, we are badly in need of of conceptual clarification.
(ii) Certainly, evolution must not be reduced to adaptation (this is a central tenet of the discussion from Gould & Lewontin’s, 1978, plea onward). If adaptation is an important, but not an exclusive concept of an evolutionary perspective, the extent to which other evolutionary processes (such as drift; Brandon, 2006; Futuyama, 1998) can be applied to human development must be examined. Although this task is still to be solved, its necessity will probably prove to be a theoretical advantage: the burden of explaining human development would have become too heavy for just one concept (adaptation).
(iii) Psychology—and developmental psychology in particular—focuses on individuals. But if evolution (according to widely held views; e.g., Futuyma, 1998; Gould, 2002; Maynard Smith, 1958; Mayr, 2001) focuses on populations – can evolution (and adaptation) be applied in a more than metaphorical sense to ontogeny of persons? Beyond a serious recognition of the “unit of selection” problem (e.g., Gould & Lloyd, 1999), any affirmative answer makes an important assumption that needs to be discussed in more detail: The thesis that the relevant unit of development (whether ontogenetic or phylogenetic) is developmental systems. Remarkably, although this approach was first formulated in evolutionary theory (Griffith & Gray, 2004; Oyama, 1985), it was taken up very early (and quite independently of an evolutionary perspective) in developmental psychology (Ford & Lerner, 1992; Thelen & Smith, 1994). It is still not central there either (many textbooks do not deal with the topic or only in passing), but it has been discussed in more detail in theoretical volumes for some time (Overton & Molenaar, 2015). If this approach can be defended, that is, if there is evolution (development) at multiple levels (Jablonka & Lamb, 2005; Sober & Wilson, 1998), then the idea that the same (not just similar) processes are operative at all levels is no longer implausible.

If humans are developmental systems (and parts of developmental systems, and consist of developmental systems), and if their development could be reconstructed—and thus: explained—in evolutionary terms, then it must be taken into account that systems do not only change linearly, but typically dynamically and in a complex way. Although the concept of dynamic systems (Feldman, 2019) was taken up early in developmental psychology (Thelen & Smith, 1994), it has so far only been taken up in relatively specialized (methodological) discussions (e.g., Boker & Wenger, 2008; see Overton & Molenaar, 2015); although it is hardly doubted on the merits, it has so far only been possible to apply it empirically, to developmental topics in narrowly defined fields (e.g., Lenzing et al., 2023). Here, the crucial challenge of the coming years will be to make such methodological (mathematical) approaches empirically useful in developmental psychology. The complex systems approach, which takes another significant step forward here (Mitchell, 2009), is even less frequently discussed—although it is recognizably worthy of attention. It is the plan of this chapter to contour the opportunities and challenges inherent in such an extension. It will be particularly fruitful not only, but especially, if it were viable to describe human ontogenesis in evolutionary terms, i.e., self-regulated.

## Conclusion

The resulting view on human development would then not only be a serious life span approach, but at the same time a biological approach, which would not be confined to a specific physical realization (i.e., would not have to presuppose certain assumptions about, for example, genetic or neurological processes). It is precisely the theoretical application of the adaptation concept that allows for a structural and processual understanding of development that is at the same time universal and not only takes individuality into account but also explains it. This view might be called “individual evolution”. Among the theoretical challenges of such an approach will be the defense of a non-reductionist psychology that takes biological processes seriously without reducing psychology to physiology. Central in this context is the thesis that the special potential of the theory of evolution lies in the abstract theory, not (only) in the precise physiological description of the way humans are factually realized (the theory of evolution has gained in plausibility since 1859, although Darwin's idea of how heredity is realized in humans and other animals was entirely incorrect).

In order to apply the concept of adaptation to human ontogeny in this theoretical and abstract manner (and not just use it metaphorically as an illustration), the question must be answered how information transfer (which in phylogenetic evolution in the sense of inheritance is one of the conditions of adaptation and thus evolution) can be thought of intraindividually, especially in a way that simultaneously ensures both variation and retention. Since the mid-twentieth century, evolutionary theory has very largely settled on the idea that in evolutionary dynamics this transgenerational transfer of information (including a sufficiently small but at the same time sufficiently large amount of unsystematic variation) is realized via genes encoded in DNA that is retained and used in the cells of all living organisms. The application of the adaptation concept to other (i.e., individual) developmental dynamics implies a comparably functional realization of horizontal (learning) and vertical (inheritance) information transfer.

It is perhaps the most difficult challenge for the envisioned application of adaptation to human ontogeny that such a concept of transfer of information must be defended against the numerous and serious criticisms that several approaches to applying evolutionary theory to non-biological entities (especially culture: Boyd & Richerson, 2005; Distin, 2011) have been subjected to. Prototypical of this discussion is the ambitious concept of “memes” (originally proposed by Dawkins, 1976; see Aunger, 2000; Blackmore, 1999; Distin, 2005), which (although the term has endured) has so far failed to prove scientifically fruitful. To prepare a future solution, it will be certainly necessary to apply some aspects of modern information theory (for a smart introduction: Gleick, 2011) to psychological content.

The quest for such an integrative application and perspective, however, might be worth the effort. The promise of this application of adaptation to human development could be (1) an integration of productive theories of human development (e.g., Piaget, Erikson, Havighurst, Bowlby, Marcia), (2) an integration of various approaches from a lifespan perspective (Baltes, 1997; Baltes & Baltes, 1990; Brandtstädter, 2006; Brandtstädter & Rothermund, 2002; Heckhausen et al., 2010, 2019; Ford & Lerner, 1992; Thelen & Smith, 1994, to name a few), (3) a conceptual framework for current issues (e.g., resilience; Greve & Staudinger, 2006; Leipold & Greve, 2009), (4) an integration of both micro-processes and macro-processes of change (Coping, Learning) into one concept of development, (5) the final extinction of intellectual zombies (e.g., a fruitless „nature-nurture “ dichotomy), and (6) the adoption of a *conceptual* evolutionary psychology perspective without using non-plausible assumptions of evolutionary psychology (such as the „massive modularity hypothesis “; Buller, 2005).

An evolutionary perspective on human development might pave the way not only to understand why we develop in the first place, and not only to better understand the current and postponed functionality of different developmental steps, but also to be able to explain how development happens, how it is controlled. We do need theory in (developmental) psychology (“No science can be ‘empirical’ only”; Valsiner, 2000, p. 2). That is why it is worth looking for an abstract and integrative theory of development.
